# Characterization of temporal expression of immune genes in female locust challenged by fungal pathogen, *Aspergillus* sp.

**DOI:** 10.3389/fimmu.2025.1565964

**Published:** 2025-04-28

**Authors:** Muhammad Saad Waqas, Xiao Xu, Pengfei Zhang, Jin Guo, Shaojing Hu, Yinwei You, Long Zhang

**Affiliations:** ^1^ Institute of Plant Protection, Shandong Key Laboratory for Green Prevention and Control of Agricultural Pests, Shandong Academy of Agricultural Sciences, Jinan, Shandong, China; ^2^ Hebei Provincial Jujube Kernel Utilization Technology Innovation Center, Department of Chemical Engineering and Biotechnology, Xingtai University, Xingtai, China

**Keywords:** innate immune, immune genes, temporal expression patterns, female locust (*Locusta migratoria*), infection, fungal pathogen (*Aspergillus oryzae*)

## Abstract

**Introduction:**

The innate immune system provides defense against invading pathogens in insects and mammals.

**Methods:**

We conducted transcriptomic analyses of the locust *Locusta migratoria* under *Aspergillus oryzae* infection to clarify temporal variation in its molecular immune response.

**Results:**

We found that fat body cells and hemocytes play different roles in the immune response of locusts to *Aspergillus* infection at different time points after inoculation, and melanization was the main process underlying the immune response of female locusts. Most pattern recognition receptors (PRR) genes were up-regulated in fat body cells and down-regulated in hemocytes from 24 h to 72 h after inoculation. This means that fat body cells, but not hemocytes, would be able to precisely recognize invading pathogens. Most serine protease inhibitors (SERPINs) genes and clip domain serine proteinase (CLIP) genes were up-regulated in fat body cells. However, most *SERPINs* were down-regulated in hemocytes, which indicated that serine proteinases may be inhibited to activate downstream reactions involving the prophenoloxidase (PPO), peroxidase (POD), and Toll pathways. Most lysozymes, PPOs, and peroxiredoxin (PRDX) are effectors that were up-regulated in fat body cells 24 h after inoculation but down-regulated 48 h and 72 h after inoculation. Similar patterns were observed for effectors in hemocytes, which indicates that locust immune genes expression was suppressed by *A. oryzae* 72 h after inoculation, and might result in the weak melanization of locusts in response to *Aspergillus* infection.

**Discussion:**

Our findings enhance our understanding of insect–fungi interactions, as well as have implications for the development of more effective microbial control strategies for the management of locust populations.

## 1 Introduction

The innate immune system of insects and mammals protects hosts against invading pathogens. Non-specific innate immune responses involve many areas of the body and play crucial roles in the prevention of disease and infections (1). There are similarities between the insect immune system and mammalian innate immune system and because the immune system in insects is free of some of the disadvantages of the mammalian immune system, many insects are used in studies of the innate immune system. Some of these insects include *Drosophila melanogaster* (2, 3), *Galleria mellonella* (4, 5), and *Bombyx mori* (6).

Several groups of genes and their pathways have been identified in insect innate immune systems. Pattern recognition receptors (PRRs), effectors, and Toll and IMD pathways are the main immune pathways in insects. PRRs such as peptidoglycan recognition protein (PGRP), β-1,3-glucan binding protein (βGBPs), Calcium-dependent (C-type) lectins, and scavenger receptors (SRs) recognize pathogen infections (7). Toll and Toll-like receptors are initiated through these PRRs and by a cascade of serine protease inhibitors (SERPINs); they play a key role in the development, adhesion, and immune responses of insects (8–10). Antimicrobial effectors are induced via the Toll and IMD (immune deficiency) pathways, as well as by the Janus kinase/signal transducer and activator of transcription (JAK-STAT) pathways to protect hosts against infection (11, 12). The fat body and hemolymph are involved in the humoral immune process, and they play different roles against pathogens (13, 14). The fat body is the main organ responsible for the synthesis of hormones, immune-related proteins, energy compounds, and pheromones (15). Various compounds that are produced in fat body are released in the hemolymph (16); the hemolymph thus plays a key role in humoral defense.

Immune responses to pathogen infections vary among insect species (17). *Metarhizium* spp. and *Beauveria* spp. synthesize destruxins and oosporein, respectively, to inhibit prophenoloxidase (PPO) activity and down-regulate the expression of antifungal peptides (18, 19). *M. anisopliae* and *M. robertsii* secrete more proteases, chitinases, G protein-coupled receptors, and detoxifying enzymes than *M. album* and *M. acridum* (18). Mosquito genomes encode nine to ten POs with different functions (20–22). Previous studies have shown that holometabolous insects, such as *Lepidoptera*, *Coleoptera*, and *Drosophila*, produce antimicrobial peptides (AMPs) through the Toll pathway after infection with fungal pathogens, and AMPs in hemimetabolous pea aphids in the order Hemiptera are produced through the JNK pathway after infection with bacterial pathogens (23). In locusts, many immune-related genes have been identified (24, 25), and Toll and IMD pathways are activated in *L. migratoria* after infection with *Metarhizium acridum* (25). However, few studies have examined the molecular immune responses of both the fat body and hemolymph over time in insects (25).

Invasive aspergillosis is a severe fungal infection caused by *Aspergillus* species in humans, and most studies of the immune response against *Aspergillus* have focused on mammals (26). The humoral immune response has been poorly studied. The virulence of medically important pathogens has been studied in various insects because of the similarities of insect immune responses with mammalian innate immune responses (27).

*Aspergiullus oryzae* XJ1 has recently been identified to be highly virulent against *L. migratoria* and other species of grasshoppers (28, 29). Study of the molecular immune mechanism of locusts against *A. oryzae* may help promote the development of new biocontrol agents for the management of locust plagues and provide insights into the innate humoral immune response in humans. Here, we conducted transcriptomic analysis and characterized temporal variation in the expression of immune genes in female locust fat body cells and hemocytes under *A. oryzae* challenge.

## 2 Materials and methods

### 2.1 Locust rearing

Locusts, *Locusta migratoria manilensis* (Orthoptera: Acrididae) were obtained from Institute of Plant Protection, Shandong Academy of Agricultural Sciences. Fifth-instar nymphs were reared in cylindrical steel cages (diameter 15 cm × height 40 cm) with 25-35 locusts per cage. Fresh wheat seedlings were provided as a food source. Observations were made daily to determine the timing of the molting of the fifth-instar nymphs into adults. Newly molted adults were collected and reared in separate cages. All female adults were reared for 2 ± 1 days at 25 ± 1°C with 55 ± 10% relative humidity and a 15 h/9 h light/dark photoperiod.

### 2.2 *A. oryzae* strains and *L. migratoria* inoculation

*A. oryzae* XJ1 was provided by the Key Laboratory for the Biological Control of Pests of the Ministry of Agriculture, China Agricultural University. The fresh conidia powder was cultured on potato dextrose agar plates at 26.0 ± 1.0°C. The conidia germination rate was 64.25 ± 5.8% (mean ± SEM, n = 4) after 24 h of incubation under standardized conditions, indicating robust conidia viability. A conidial suspension with a concentration of 1 × 10^7^ conidia mL^-1^ was prepared by suspending conidia in sterile double-distilled water containing 0.3% (vol/vol) Tween-80. The concentration of the conidia was quantified using a Neubauer hemocytometer under a light microscope to ensure accuracy. Healthy adult females were fully immersed in the prepared conidial suspension about 2 seconds to ensure consistent exposure according to previous studies (28, 29). Control adults were inoculated with 0.3% (vol/vol) Tween-80 solution. After treatment, all treated locusts were reared in clear plastic boxes. Adults were housed separately in groups of 3 ± 1 adults in individual clear plastic boxes with a round top (diameter, 14 cm), round bottom (diameter, 9 cm), and height of 14 cm. All treated and control adults were reared for 24 ± 2 h, 48 ± 2 h, and 72 ± 2 h at 25 ± 2°C with 55 ± 10% relative humidity and a 15 h/9 h light/dark photoperiod.

### 2.3 RNA extraction and sequencing

Female adults were collected from the boxes for RNA extraction. The arthrodial membrane of the hindlegs of treated and control adults was swabbed and air-dried for 15 min for the collection of hemolymph. After cutting the hindleg, female adult hemolymph was collected using a micropipette and transferred to Trizol reagent (Invitrogen, U.S.A). Fat bodies were collected by dissecting the adults on a waxed Petri dish. All collected fat bodies were transferred to Trizol reagent for RNA extraction. RNA was extracted using the Trizol Reagent following the manufacturer instructions. Briefly, the sample was homogenized in 1 mL of Trizol Reagent at room temperature for 5 min. Next, 200 μL of trichloromethane was added and mixed well; the mixture was then left to stand at room temperature for 2 min and centrifuged at 12,000 rpm and 4°C for 15 min. The supernatant was then removed. Subsequently, 500 μL of isopropyl alcohol was added and mixed well; the mixture was then left at room temperature for 10 min and centrifuged at 12,000 rpm and 4°C for 10 min; the precipitate was retained. One mL of 75% ethanol was washed twice (centrifuged at 7,500 rpm and 4°C for 5 min); the precipitate was retained, and an adequate amount of RNase-free water was added after fully drying. The quantity and quality of RNA samples were examined using a Nanodrop spectrophotometer (Thermo Scientific, U.S.A.). A total of four replicates of treated individuals and control individuals were performed for the female fat body samples at each time interval. Fat bodies were collected from 6–10 female adults. A total of four boxes were considered one replicate in the fat body extraction experiment. A total of four replicates of treated individuals and control individuals were performed for the female hemolymph samples 24 h and 72 h after inoculation while three replications of treated and four replications of control were performed 48 h after inoculation. Hemolymph was collected from 10–15 female adults. A total of five boxes were considered one replicate for hemolymph extraction.

mRNA was purified from total RNA using poly-T oligo-attached magnetic beads. After fragmentation, the first-strand cDNA was synthesized using random hexamer primers followed by second-strand cDNA synthesis. The library was ready after end-repair, A-tailing, adapter ligation, size selection, amplification, and purification. The library was checked using a Qubit 2.0 Fluorometer (Thermo Scientific, U.S.A.) and real-time PCR for quantification, and an Agilent 2100 bioanalyzer was used to characterize the size distribution. After library quality control, different libraries were pooled based on the effective concentration (1.5 nM) and targeted amount of data; they were then subjected to Illumina sequencing (a platform provided by Novogene company, China). The basic principle of the sequencing process is “Sequencing by Synthesis,” where fluorescently labeled dNTPs, DNA polymerase, and adapter primers are added to the sequencing flow cell for amplification. The sequencer captures these fluorescence signals and converts them into sequencing peaks through computer software, which yields sequence information of the target fragment.

### 2.4 Gene identification

Clean reads for subsequent analysis were obtained after filtering raw data and checking the sequencing error rate and GC content distribution. Hisat2 v2.0.5 software (Johns Hopkins University, U.S.A.) was used to quickly and accurately compare clean reads with the reference genome to obtain the reference genome’s location information. Novel transcripts were assembled using String Tie software (Johns Hopkins University, U.S.A.) and annotated using the Pfam database (European Bioinformatics Institute, U.K.).

FeatureCounts v1.5.0-p3 (The Walter and Eliza Hall Institute of Medical Research, Australia) was used to count the number of reads mapped to each gene. Analysis of differentially expressed genes across samples was performed using the DESeq2 R package (1.20.0). DESeq2 provides statistical programs to identify differentially expressed genes by modeling count data using a negative binomial distribution. The significance of differential expression was assessed through hypothesis testing, with P-values calculated based on Wald tests. P-values were subsequently adjusted using the Benjamini and Hochberg’s methods, which controls the false discovery rate (FDR) at a predefined threshold. Genes meeting the criteria of adjusted P-value ≤ 0.05 were considered different-expressed genes, and log_2_(foldchange) of the gene was calculated according to normalized reads. In this study, genes whose log_2_(foldchange) > 0 were called up-regulated genes, and whose log_2_(foldchange) < 0 were called down-regulated genes. Based on gene annotation, Immune-related genes were identified from the differentially expressed genes in control and treated populations. All genes associated with these immune-related genes were further validated using BLAST analysis on the NCBI database, and matched genes were kept. Then we calculated the FPKM of each gene based on the length of the gene and the read count mapped to this gene, and further compared the FPKM values of up/down-regulated genes between treated and control with *t*-tests, to clarify the significantly changes of their expression levels between treated and control.

### 2.5 Statistical analysis

According to the principal component analysis, the few samples that were not clustered together were deleted, and the gene expression levels of the remaining samples were compared. Differences in the FPKM values of genes between control and treated populations were analyzed using *t*-tests. The relative FPKM value of a gene was calculated based on the FPKM value of the gene in the treatment populations subtracted from the average FPKM value of the gene in the control populations. The relative FPKM values of genes varying in expression in the treated populations among time points were analyzed using one-way ANOVA followed by Tukey’s multiple comparison test with the following significance thresholds: *, p < 0.05; **, p < 0.01; ***, p < 0.001; ****, p < 0.0001. All Figures were drawn with Microsoft Excel and GraphPad Prism 7 (GraphPad Software, San Diego, CA, United States).

## 3 Results

### 3.1 Temporal variation in gene expression in *L. migratoria* fat body cells and hemocytes after exposure to *A. oryzae*

The transcriptome of female *L. migratoria* adult fat body cells and hemocytes from control and infected with *A. oryzae* were analyzed. Fungal infection induced a marked shift in the genes expression in fat body cells and hemocytes. Differential gene expression analysis revealed 1,048 DEGs, 1,891 DEGs, and 1,037 DEGs in female fat body cells 24 h, 48 h, and 72 h after inoculation, respectively. The fold change of up-regulated and down-regulated genes was higher 48 h after *A. oryzae* inoculation compared with that 24 h and 72 h after *A. oryzae* inoculation (**Table 1**). Differential gene expression analysis revealed 873 DEGs, 881 DEGs, and 840 DEGs in female hemocytes 24 h, 48 h, and 72 h after inoculation, respectively. The number of down-regulated genes in hemocytes was higher than the number of up-regulated genes (**Table 1**).

**Table 1:** Up-regulated and down-regulated genes in female fat body cells and hemocytes in three- time treatment after infection with *A. oryzae*.

Time (h)	Fat body cells		Hemocytes		Total
	Up-regulated gene	Down-regulated gene	Up-regulated gene	Down-regulated gene	
24	572	476	427	446	1921
48	934	957	217	664	2772
72	588	449	291	549	1877

### 3.2 Differential expression of immune genes

The immune response of female fat body cells and hemocytes varied over time after *A. oryzae* inoculation. The total number of immune-related genes decreased in female fat body cells over time. In fat body, the total number of up-regulated genes was higher than down-regulated ones; in contrast, in hemocytes the number of up-regulated genes was lower than down-regulated ones at 48 h and 72 h after *A. oryzae* inoculation. (**Table 2**). There were 26, 17, and 12 up-regulated immune genes in female fat body cells 24 h, 48 h, and 72 h after *A. oryzae* inoculation, respectively, and there were 6, 13, and 10 down-regulated immune genes 24 h, 48 h, and 72 h after *A. oryzae* inoculation, respectively (**Table 2**). The up-regulated genes were higher than down-regulated genes 24 h after *A. oryzae* inoculation and up-regulated genes were lower than down-regulated genes 48 h and 72 h after *A. oryzae* inoculation in female hemocytes (**Table 2**). To further elucidate the roles of differentially expressed genes in the immune responses of *L. migratoria*, we examined the expression level of the immune related genes.

**Table 2:** Total number of up-regulated and down-regulated immune related genes in female fat body cells and hemocytes after *A. oryzae* infection.

Time (h)	Fat body cells		Hemocytes		Total
	Up-regulated gene	Down-regulated gene	Up-regulated gene	Down-regulated gene	
24	26	6	7	5	44
48	17	13	2	14	46
72	12	10	3	10	35

#### 3.2.1 PRRs

**Figure 1:**
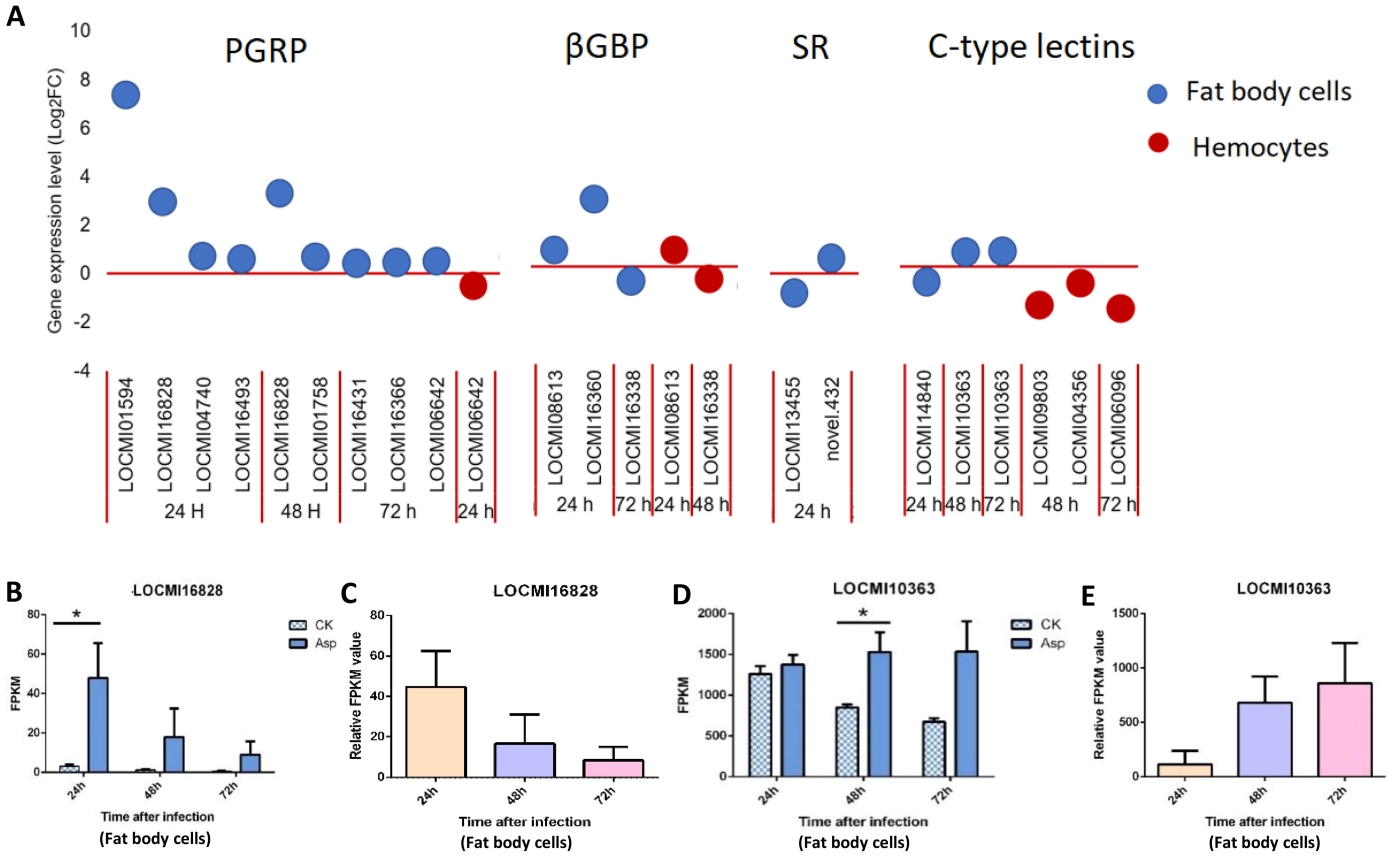
The temporal expression of pattern recognition receptor genes in female locust fat body cells and hemocytes. n=3-4. *p < 0.05. Bar, S.E.M. **(A)**, PRR up-regulated and down-regulated genes after 24 h, 48 h and 72 h inoculation with fungal pathogen, *A. oryzae* XJ1. **(B, D)**. Differential statistical analysis of expression levels of the gene in fat body between control and infected locusts. CK, control; Asp, *A. oryzae*. t-test. **(C, E)**. Statistical analysis of temporal expression levels of the same gene in fat body of infected locusts. Ordinary two-way ANOVA. Tukey’s multiple comparisons test.

In locust fat body cells, the total number of up/down-regulated PGRP genes decreased from 24 h to 72 h after *A. oryzae* inoculation; 4, 2, and 3 PGRPs were expressed 24 h, 48 h, and 72 h after *A. oryzae* inoculation, respectively, and these genes were all up-regulated. The PGRP-LB genes LOCMI01594 and LOCMI16828 were highly up-regulated in female fat body cells compared with other PGRP genes in fat body cells (**Figure 1A**). The total number of up/down-regulated βGBP genes decreased from 24 to 72 h after *A. oryzae* inoculation, and 2 and 1 βGBP genes were expressed 24 h and 72 h after *A. oryzae* inoculation in fat body cells, respectively (**Figure 1A**). The expression levels of the other PRR genes, including *PGRP*, *βGBP*, *SRs*, and *C-type lectins* in fat body cells, did not significantly vary over time.

In hemocytes, the total number of up/down-regulated PRR genes was 6, and this was less than the number of fat body cell genes. In contrast to fat body cell genes, no genes were highly up-regulated in hemocytes, but two C-type lectin genes, LOCMI09803 and LOCMI06096, were highly down-regulated 48 h and 72 h after *A. oryzae* inoculation (**Figure 1A**), indicating that these two genes might be inhibited by *A. oryzae*.

We also analyzed the expression of these same genes expressed at different time intervals. The PGRP-LB gene LOCMI16828 was expressed in fat body cells 24 h and 48 h after *A. oryzae* inoculation, and its expression 24 h after inoculation was significantly higher in treated individuals than in control individuals (**Figure 1B**); however, the relative expression of this gene continuously decreased over time in the fat body cells of treated adults (**Figure 1C**). The expression of the C-type lectin gene LOCMI10363 was significantly higher in treated adults than in control adults 48 h after inoculation (**Figure 1D**), and its relative expression increased over time in the treated individuals (**Figure 1E**).

#### 3.2.2 Serine protease, SERPINs and clip domain serine proteinase (CLIP)

**Figure 2:**
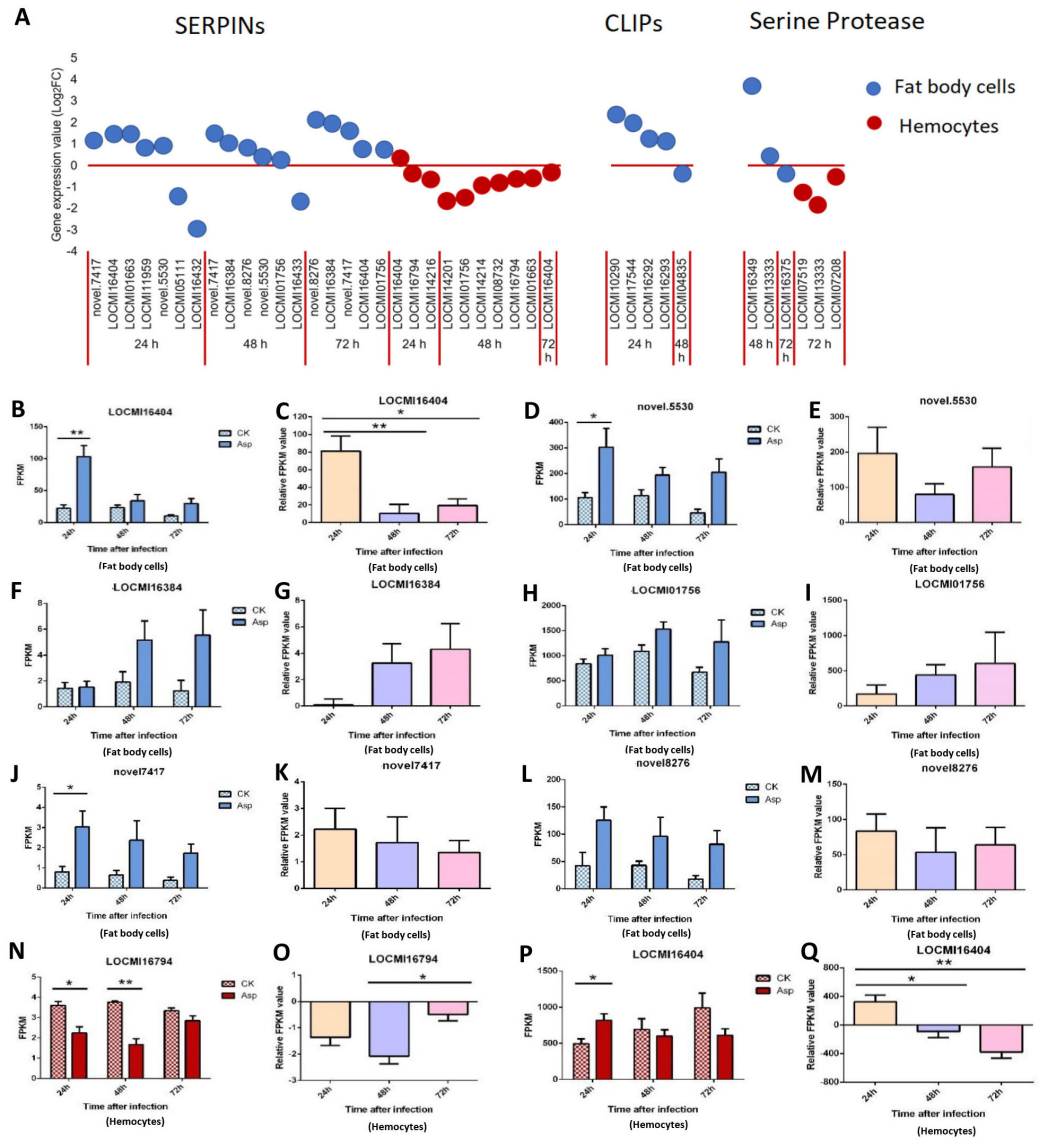
The temporal expression of the genes of SERPINs, CLIP and serine proteases in female locust fat body cells and hemocytes. *p < 0.05. **p < 0.01. n=3-4. Bar, S.E.M. **(A)**, The dynamics of the SERPINs, Serine proteases and CLIPs in female locust fat body and hemocytes after 24 h, 48 h and 72 h inoculation with fungal pathogen, *A. oryzae* XJ1. **(B, D, F, H, J, L)**, Differential statistical analysis of expression levels of the gene in fat body, and **(N, P)**, in hemocytes between control and infected locusts. t-test. **(C, E, G, I, K, M, O)**, Statistical analysis of temporal expression levels of the same gene in fat body of infected locusts, and **(O, Q)**, in hemocytes. CK, control; Asp, *A. oryzae*. Ordinary two-way ANOVA. Tukey’s multiple comparisons test.

In fat body cells, the total number of up/down-regulated SERPIN genes decreased; 7, 6, and 5 genes were expressed in fat body cells 24 h, 48 h, and 72 h after *A. oryzae* inoculation, respectively. There were 2, 1, and 0 down-regulated SERPIN genes in fat body cells 24 h, 48 h, and 72 h after inoculation, respectively; there were five up-regulated genes 24 h, 48 h, and 72 h after inoculation (**Figure 2A**). All CLIP genes were up-regulated 24 h after inoculation and down-regulated 48 h after inoculation; they were expressed only in female fat body cells and not in the hemocytes.

In hemocytes, the number of up/down-regulated SERPIN genes were 10 at all three time points, and number of SERPIN genes was highest 48 h after inoculation. Among the 10 genes, 9 were down-regulated, and the expression patterns of these genes in hemocytes and fat body cells differed (**Figure 2A**). Two serine protease genes were up-regulated 48 h after inoculation and one down-regulated 72 h after inoculation in fat body cells while all serine protease was down-regulated in hemocytes 72 h after inoculation (**Figure 2A**).

The gene LOCMI16404 was expressed in both fat body cells and hemocytes, and it was up-regulated in fat body cells 24 h, 48 h, and 72 h after inoculation in treated adults compared with the findings in control adults (**Figure 2B**). The expression of this gene was down-regulated in hemocytes 48 h and 72 h after inoculation in treated adults compared with the findings in control adults (**Figure 2P**). The relative expression level of *LOCMI16404* was significantly higher 24 h after inoculation than after 48 h and 72 h of inoculation in fat body cells and hemocytes (**Figures 2C, Q**).

The expression level of *novel.5530* was significantly higher in treated individuals than in control individuals 24 h after inoculation, but no significant difference was observed between treatments and the control at the other two time points (**Figures 2D, E**). The expression of the SERPIN genes, LOCMI16384, and LOCMI01756, increased over time in fat body cells, but there were no significant differences between treated and control adults or between adults at different time points (**Figures 2F–I**). Expression levels of *novel.7417* and *novel.8276* were higher in treated female fat body cells than in control female fat body cells (**Figures 2J, L**), and the relative expression of treated female adults was higher in female fat body cells 24 h after inoculation than 48 h and 72 h after inoculation (**Figures 2K, M**). The SERPIN gene LOCMI16794 was significantly down-regulated in female hemocytes 24 h and 48 h after inoculation in treated individuals compared with the findings in control individuals however 72 h after inoculation, no difference was observed in the expression of this gene between infected and control individuals, and this gene was down-regulated in treated adults (**Figures 2N, O**).

#### 3.2.3 Toll pathway and JAK/STAT pathway

**Figure 3:**
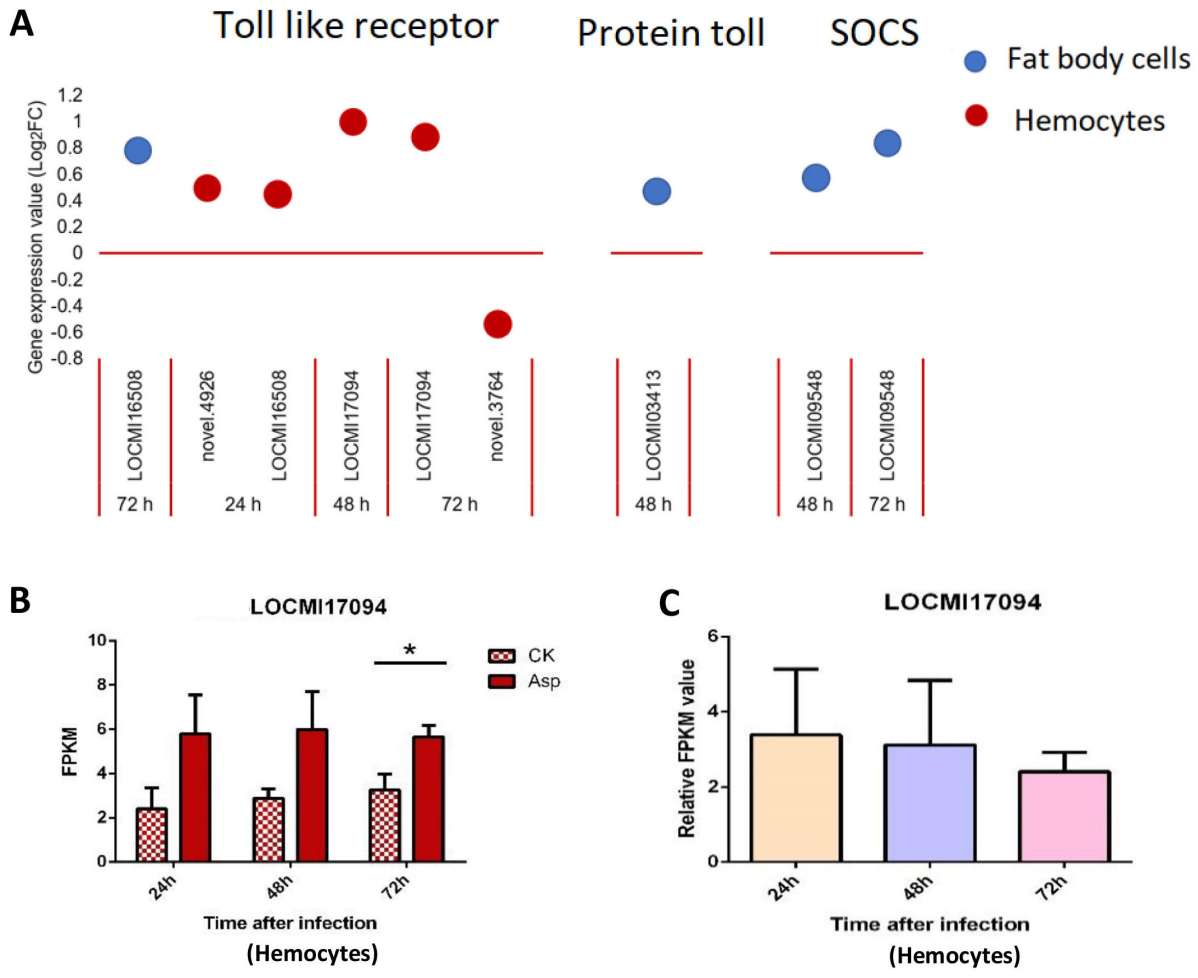
The temporal expression of the genes of Toll-pathway and JAK/STAT in female locust fat body cells and hemocytes. n=3-4. Bar, S.E.M. **(A)**, The dynamics of the Toll-pathway and JAK/STAT genes in female locust fat body cells and hemocytes after 24 h, 48 h and 72 h inoculation with fungal pathogen, *A oryzae* XJ1. **(B)**, Differential statistical analysis of expression levels of the gene in hemocytes between control and infected locusts. t-test. *p < 0.05. **(C)**, Statistical analysis of temporal expression levels of the same gene in hemocytes of infected locusts. CK, control; Asp, *A oryzae*. Ordinary two-way ANOVA. Tukey’s multiple comparisons test.

Only one *Toll-like receptor* was up-regulated in female fat body cells; in hemocytes, 4 *Toll-like receptors* were up-regulated and 1 was down-regulated compared with the findings in the control (**Figure 3A**). There was only one Protein toll gene that was up-regulated 48 h after inoculation; two suppressor of cytokine signaling (SOCS) genes in the JAK/STAT pathway were up-regulated 48 h and 72 h after inoculation in female fat body cells (**Figure 3A**).

The expression of the Toll-like receptor gene LOCMI17094 significantly differ between treated and control females 72 h after inoculation, and no significant difference was observed between treated and control females 24 h and 48 h after inoculation; no significant differences in the expression of this gene were observed between treated adults at different time points (**Figures 3B, C**).

#### 3.2.4 AMPs, PPO, prophenoloxidase-activating factor (PPAF) and peroxidase (POD)

**Figure 4:**
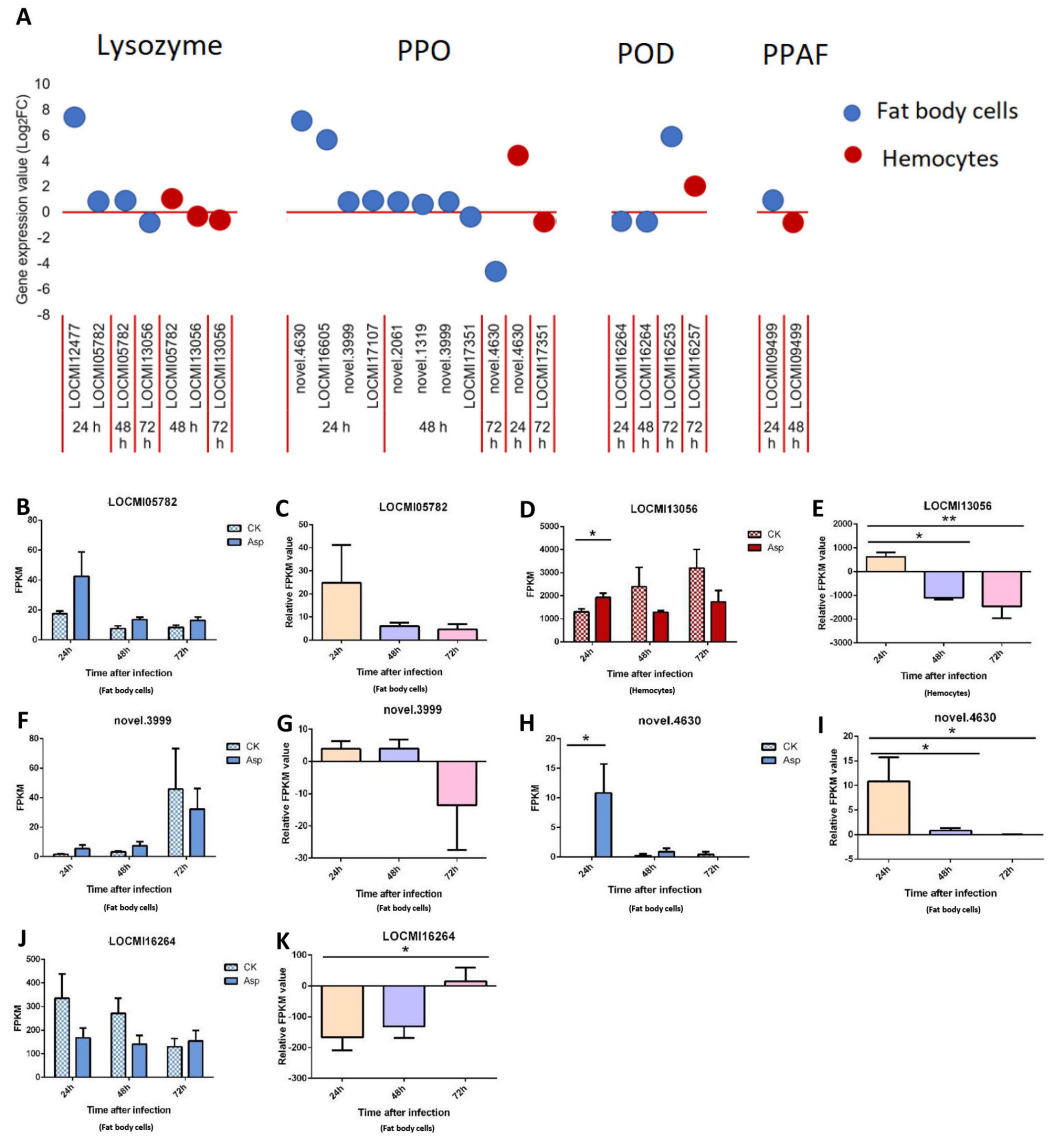
The temporal expression of the genes in effector group in female locust fat body cells and hemocytes. n=3-4. *p < 0.05. **p < 0.01. Bar, S.E.M. **(A)**, The dynamics of the effector genes in female locust fat body and hemocytes after 24 h, 48 h and 72 h inoculation with fungal pathogen, *A. oryzae* XJ1. **(B, F, H, J)**, Differential statistical analysis of expression levels of the gene in fat body, and **D,** in hemocytes between control and infected locusts. t-test. **(C, G, I, K)**, Statistical analysis of temporal expression levels of the same gene in fat body of infected locusts, and **(E)**, in hemocytes. CK, control; Asp, *A. oryzae*. Ordinary two-way ANOVA. Tukey’s multiple comparisons test.

In response to *A. oryzae* infection, four lysozyme genes were up- and down-regulated in fat body cells, and this was higher than the number of genes that were differentially expressed in hemocytes. The lysozyme gene LOCMI2477 was the most highly up-regulated among all lysozyme genes, and its Log_2_FC value (>8) was highest 24 h after inoculation with *A oryzae*; the Log_2_FC values of the other three lysozyme genes in fat body cells were less than 1.5 (**Figure 4A**).

In response to *A oryzae* infection, 9 and 2 PPO genes were up/down-regulated in fat body cells and hemocytes, respectively. Four and three PPO genes were up-regulated 24 h and 48 h after inoculation, respectively, and one PPO gene was down-regulated 72 h after inoculation. The two most highly up-regulated genes in female fat body cells 24 h after inoculation with *A oryzae* were *novel.4630* and *LOCMI16605*. Two PPO genes were differentially regulated in hemocytes: one was down-regulated, and other was down-regulated (**Figure 4A**). *PPAF* was up-regulated in fat body cells 24 h after inoculation and up-regulated in hemocytes 48 h after inoculation (**Figure 4A**).

A total of four POD genes were up/down-regulated in both fat body cells and hemocytes. Two were up-regulated and one was down-regulated in fat body cells, and only one POD gene was up-regulated in hemocytes.

The expression level of the lysozyme gene LOCMI05782 was higher in treated adults than in control adults, albeit these differences were not significant; the expression of this gene among treated individuals decreased over time (**Figures 4B, C**). However, the expression of the lysozyme gene LOCMI13056 in hemocytes significantly differed in treated and control adults 24 h after inoculation, and its expression in treated adults at 24 h significantly differed from that at 48 h and 72 h (**Figures 4D, E**). The expression of the PPO gene novel.3999 did not significantly differ in control and treated adults (**Figures 4F, G**). The expression of the PPO gene novel.4630 in fat body cells was significantly higher 24 h after inoculation in treated adults than in control adults (**Figure 4H**), and the relative expression level of this gene was also significantly higher among treated adults 24 h after inoculation than that at 48 h and 72 h after inoculation (**Figure 4I**). The expression of the POD gene LOCMI16264 did not significantly differ between treated and control adults (**Figure 4J**) and the relative expression level of this gene was significantly differed between treated adults 24 h and 72 h after inoculation (**Figure 4K**).

#### 3.2.5 Antioxidant enzymes

**Figure 5:**
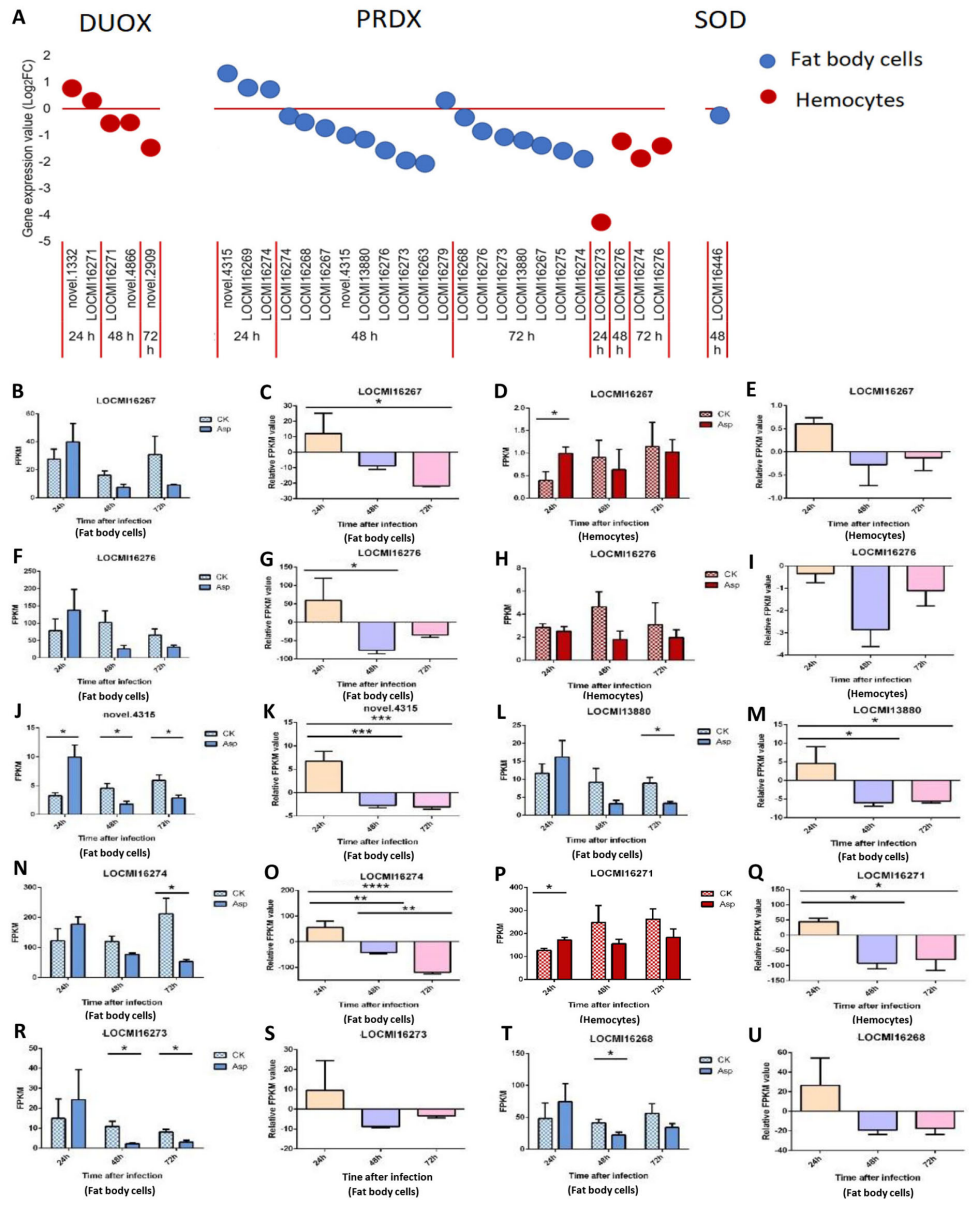
The temporal expression of the genes of effectors in female locust fat body cells and hemocytes. n=3-4. *p < 0.05. **p < 0.01. ***p < 0.001. Bar, S.E.M. **(A)**, The dynamics of the effector genes in female locust fat body cells and hemocytes after 24 h, 48 h, and 72 h inoculation with the fungal pathogen, *A. oryzae* XJ1. **(B, D, F, J, L, N, R, T)**, Differential statistical analysis of expression levels of the gene in fat body, and **(D, H, P)**, in hemocytes between control and infected locusts. t-test. **(C, G, K, M, O, S, U)**, Statistical analysis of temporal expression levels of the same gene in fat body of infected locusts, and **(E, I, Q)**, in hemocytes. CK, control; Asp, *A. oryzae*. Ordinary two-way ANOVA. Tukey’s multiple comparisons test.

The expression of all Dual Oxidase (Duox) genes was altered only in hemocytes. Duox genes were up-regulated 24 h after inoculation and down-regulated 48 h and 72 h after inoculation (**Figure 5A**). *LOCMI16271* was up-regulated 24 h after inoculation and down-regulated 48 h after inoculation.

A total of 23 peroxiredoxin (PRDX) genes were up- and down-regulated in fat body cells and hemocytes (**Figure 5A**). The novel gene (novel.4315) was up-regulated 24 h after inoculation and down-regulated 48 h after inoculation; *LOCMI16274* was up-regulated 24 h after inoculation and down-regulated 48 h and 72 h after inoculation. *LOCMI16267* and *LOCMI13380* were down-regulated 48 h and 72 h after inoculation in fat body cells. The PRDX genes LOCMI16276 and LOCMI16274 were down-regulated 48 h and 72 h after inoculation in fat body cells and hemocytes. The expression of only one Superoxide dismutase (SOD) gene was altered in female fat body cells following inoculation (**Figure 5A**).

There were no significant differences in the expression levels of the PRDX gene LOCMI16267 in treated and control adults at all three time points in fat body cells, but significant differences in hemocytes were observed 24 h after inoculation (**Figures 5B, D**). However, the relative expression levels of this gene 24 h after inoculation were higher than that after 48 h and 72 h inoculation in these two tissues (**Figures 5C, E**). In fat body, the expression level of PRDX gene LOCMI16276 showed a transient increase 24 h after inoculation, followed by a decline 48 h and 72 h after inoculation (**Figure 5G**). However, these changes were not statistically significant compared to the control group (**Figure 5F**). In hemocytes, the expression level of *LOCMI16276* decreased at all time points after inoculation, though the reductions were not significant relative to the control (**Figures 5H, I**). Significant differences in the expression of the PRDX gene novel.4315 was observed between treated and control adults 24 h and 48 h after inoculation (**Figure 5J**), and significant differences in the expression of *LOCMI13880* were observed 72 h after inoculation in fat body cells (**Figure 5L**). The relative expression levels of the PRDX genes novel.4315 and LOCMI13880 significantly higher 24 h after inoculation than 48 h and 72 h after inoculation (**Figures 5K, M**). The expression level of the PRDX gene LOCMI16274 was significantly lower in treated adults than in control adults 72 h after inoculation (**Figure 5N**), and significant differences in the relative expression level of this gene were observed among the all-time points (**Figure 5O**). The expression of the Duox gene LOCMI16271 24 h after inoculation was significantly higher in treated adults than in control adults, and significant differences in the relative expression level of this gene was significantly higher 24 h than 48 h and 72 h after inoculation (**Figures 5P, Q**). The expression of PRDX genes LOCMI16273 and LOCMI16268 significantly differ 48 h after inoculation between treatments and the control (**Figures 5R, T**). The relative expression levels of the PRDX genes LOCMI16273 and LOCMI16268 were higher 24 h after inoculation than 48 h and 72 h after inoculation (**Figures 5S, U**).

## 4 Discussion

In insects, SERPINs play a critical role in suppressing the serine protease cascade, thereby negatively regulating the activation of PPOs and Toll signaling pathways. This regulation prevents the overexpression of AMPs and excessive melanization, which could harm host (30, 31). For example, in *Drosophila*, SERPIN Spn27Ac inhibits PPO activation and suppresses melanization (32). In our study, the expression of most *SERPINs* and *CLIPs* was up-regulated in fat body cells (**Figure 2A**), which was similar to the regulation of *SERPIN* observed after bacterial infection in silkworm (33), In contrast, the expression of most *SERPINs* was down-regulated in hemocytes (**Figure 2A**), suggesting tissue-specific regulation of immune responses. This differential expression pattern highlights the complex and context-dependent roles of SERPINs in modulating immune pathways across different tissues.

In insects, the Toll pathway is essential that mediates resistance to pathogenic fungi (34). This pathway is typically initiated by the recognition of pathogen-associated molecular patterns (PAMPs) through PRRs, followed by the activation of serine protease cascades (35, 36). The canonic components of the Toll pathway include the extracellular cytokine Spätzle, the Toll-like receptor (TLR), the adaptor proteins Tube and MyD88, the kinase Pelle, the inhibitory protein Cactus (the *Drosophila* homolog of IκB), and the trans activators Dorsal and Dif (37). Furthermore, survival assays have revealed that Toll pathway’s response to entomopathogenic fungi is mediated by an extracellular proteolytic cascade involving the CLIPs, Persephone and the inhibitory serpin Necrotic (38, 39). The Spätzle, a key ligand in the insect Toll pathway, activates downstream immune responses by binding to Toll receptors (40). In our study, although we identified four Spätzle genes in the transcriptome of the treatment group, their expression levels showed no significant differences compared to the control group, indicating that Spätzle was not regulated within 72 h of *A. oryzae* infection (**Supplementary Figure S1**).

Studies have demonstrated that the deletion of any of these components (except for Cactus and Dorsal) in this pathway results in a similar immune-deficient phenotype, characterized by the absence of immune genes expression, including antifungal peptide genes, and increased susceptibility to fungal and Gram-positive bacterial infections (40, 41). No significant up-regulation or down-regulation of MyD88, Cactus, or antifungal peptide genes were observed in our study. However, the TLR genes were observed to be up/down-regulated (**Figure 3A**), sequence alignment revealed that *LOCMI16508*, *novel. 4926*, and *novel. 3764* all belong to the TLR-2 family, which can be directly activated by chitin present in the fungal cell wall (42); and *LOCMI17094* belongs to the TLR-7 family, which is primarily associated with antiviral immunity and also plays a role in the immune response caused by fungal infections (43, 44).

Although these changes were observed, considering the absence of significant changes in the expression of key Toll pathway components and the lack of downstream immune gene activation, we suggested that the Toll immune pathway was not fully activated 72 h after inoculation with *A. oryzae*. This suggests that other immune mechanisms may be primarily responsible for the observed response at this time point. While our data implicate this hypothesis, we acknowledge that this study did not directly measure key regulatory elements such as Spätzle, a critical upstream protease-activated ligand required for Toll pathway activation in insects. In future work, we will quantify the levels of Spätzle and other key genes in infected hosts to clarify this mechanism.

Melanization, a critical arthropod defense mechanism, plays a pivotal role in processes such as wound healing, encapsulation of pathogens, sequestration of microorganisms, and the production of toxic intermediates that can kill invading microbes (45–47). The melanization cascade is typically triggered by tissue injury or through the recognition of microbial ligands, such as peptidoglycan, β-1,3-glucan binding protein, and lipopolysaccharide (LPS) by PRRs (48–51). Melanization is mediated by the phenoloxidase (PO), which catalyzes the oxidation of mono- and diphenols to orthoquinones. These orthoquinones subsequently polymerize non-enzymatically to form melanin. Enzymatically inactive PPO is cleaved into active PO by prophenoloxidase-activating enzymes, and the activation of PPO is tightly regulated. The CLIPs modulate melanization by regulating PPO activation, a process that is further controlled by SERPINs (52). Survival assays have demonstrated that PPO mutant increased susceptibility to Gram-positive bacterial and fungal infections, underscoring the non-redundant role of melanization in immune defense against fungal pathogens (52).

Notably, we identified up-regulation of *PGRPs*, *CLIPs*, *SERPINs*, and *lysozyme*s, which are known to play critical roles in the melanization reaction through the one Toll-like receptor (Toll-7) and the Protein toll (**Figures 1A**, **2A**). The lysozyme genes (LOCMI13056, LOCMI05782) and PPO genes (novel.4630, LOCMI17351) were appeared together both in fat body cells and hemocytes (**Figure 4A**). However, the functional roles of these genes in the two tissues remain unknown and warrant further investigation. We further observed a dynamic regulation of immune effectors, including *lysozymes*, *PPOs*, and *PRDXs*, in the fat body cells and hemocytes of locusts following *A. oryzae* inoculation. Specifically, most of these effectors, particularly *lysozyme* (*LOCMI13056*), *PPO2* (*novel. 4630*), and *PRDX* (*novel. 4315*), were significantly up-regulated 24 h post-inoculation but showed down-regulation by 72 h (**Figures 4A, D, E, H, I**, **5A, J, K**). This temporal pattern suggests an initial robust immune response, characterized by a strong melanization reaction in *L. migratoria* 24 h after *A. oryzae* inoculation. However, by 72 h, *PPO*, such as *PPO2*, was down-regulated, indicating a reduction in melanization activity, which likely diminished the defense response against fungal pathogens (53). This temporal pattern is different in other insect species, such as most *PPOs* or *lysozyme* were up-regulated after fungus inoculation in *C. lectularius* and honey bee (17, 54). These findings led us to hypothesize that melanization is a critical defense mechanism in locusts against *A. oryzae* during the early stages of infection, but may be inhibited by the pathogen over time, and it needs to be demonstrated by further studies.

This also suggest that *A. oryzae* suppress the locusts’ immune genes expression following infection. Although locusts initially resisted *A. oryzae* by up-regulating key immune components such as *lysozymes*, *PPOs*, and *PRDXs* 24 h after inoculation, but *A. oryzae* appeared to suppress the melanization response by inhibiting the expression levels of key immune genes, such as *PPOs*, 48-72 h after inoculation (**Figures 4A**, **5A**). The suppression of immune-related genes expression may impair locust immunity, facilitating the *A. oryzae* to establish infection for locusts. The suppression of immune-related genes could be exploited in the development of biological control strategies for locusts using *A. oryzae*. Such as since PPOs and PRDXs play key roles in defense of locust against *Aspergillus*, it would be possible to find and develop some inhibitors of these enzymes as enhancing factors for *Aspergillus* to control locusts. Our results indicated that fat body play a crucial role in defense against *Aspergillus* infection, this inspires to develop the combination use of *Aspergillus* and the pathogen, *Nosema locustae* (Protozoa: Microsporidia) whose main target is fat body, to improve the effects of controlling locusts (55), and it is supported by an recent study (56).

The innate immune systems of insects and mammals share both humoral and cellular responses, with hemocytes in insects and myeloid cells in mammals serving as key cellular mediators (57). Hemocytes, like mammalian neutrophils, target pathogens through mechanisms such as phagocytosis, encapsulation, and superoxide production (58–60). Our results implied that hemocytes in locusts play a critical role in the immune response to fungal pathogens by modulating pathogen recognition and immune signaling while preventing excessive activation. Unlike the fat body, which primarily produces AMPs and effector molecules, hemocytes exhibit fewer up-regulated *PRRs* and down-regulated specific C-type lectins and SERPIN genes, likely to control protease activity and inflammation. Hemocytes also transiently activate Duox genes to generate reactive oxygen species (ROS), which are later suppressed to avoid oxidative damage, complementing the fat body’s effector role. Locust hemocytes release C-type lectins into the hemolymph, which may mediate immune responses in the fat body just like in *B. mori* and *Tribolium castaneum* (61, 62).

**Figure 6:**
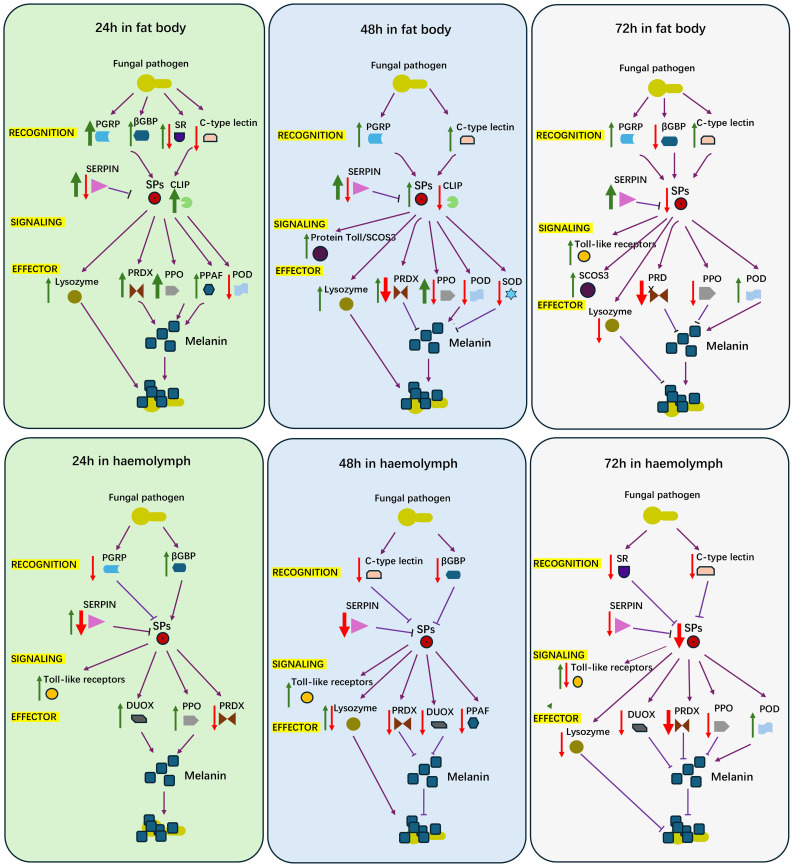
A diagram for proposed features of female locust adult humoral immune response to challenge of *Aspergillus oryzae* XJ1. Red arrow, down regulated genes; Green arrow, up regulated genes; Purple arrow, the activated relationship; Purple T bar, inhibited relationship; Think arrow, more number of genes; Thin arrow, less number of genes.

In summary, our findings highlight the dynamic interplay between fat body cells and hemocytes during the immune response to fungal pathogens in adult female locusts. The fat body drives humoral responses, including the secretion of AMPs and melanization, and serves as the primary site for immune effector production. In contrast, hemocytes play a complementary role in pathogen recognition, immune regulation, and reactive oxygen species (ROS) management, ensuring a balanced and effective immune response through their regulatory functions (**Figure 6**). However, our study was limited to analyzing the differential expression of immune genes at the transcriptome level in fat body cells and hemocytes, exploring potential immune responses. In future research, we plan to conduct qRT-PCR, gene editing of selected immune genes, and other related experiments, including chromatographic analyses of hemolymph composition. These efforts will help verify the functions of key genes in the immune response and provide a comprehensive understanding of the systemic immune mechanisms in insects, as well as their temporal and spatial dynamics during pathogen challenges.

## Data Availability

The data presented in the study are deposited in the NCBI repository, accession number PRJNA1211842, PRJNA1212177, PRJNA1212339, PRJNA1212589, PRJNA1212757, PRJNA1212830, PRJNA1213078, PRJNA1213165, PRJNA1213310, PRJNA1213846, PRJNA1213978, PRJNA1214046, PRJNA1214369, PRJNA1214481, PRJNA1214552.
