# Supplementary material for: Characterization of temporal expression of immune genes in female locust challenged by fungal pathogen, Aspergillus sp
Source: Front Immunol. 2025 Apr 28;16:1565964. doi: 10.3389/fimmu.2025.1565964 (PMC12066608; doi:10.3389/fimmu.2025.1565964)
Supplement: Supplementary file 1 [file DataSheet1.docx]

**
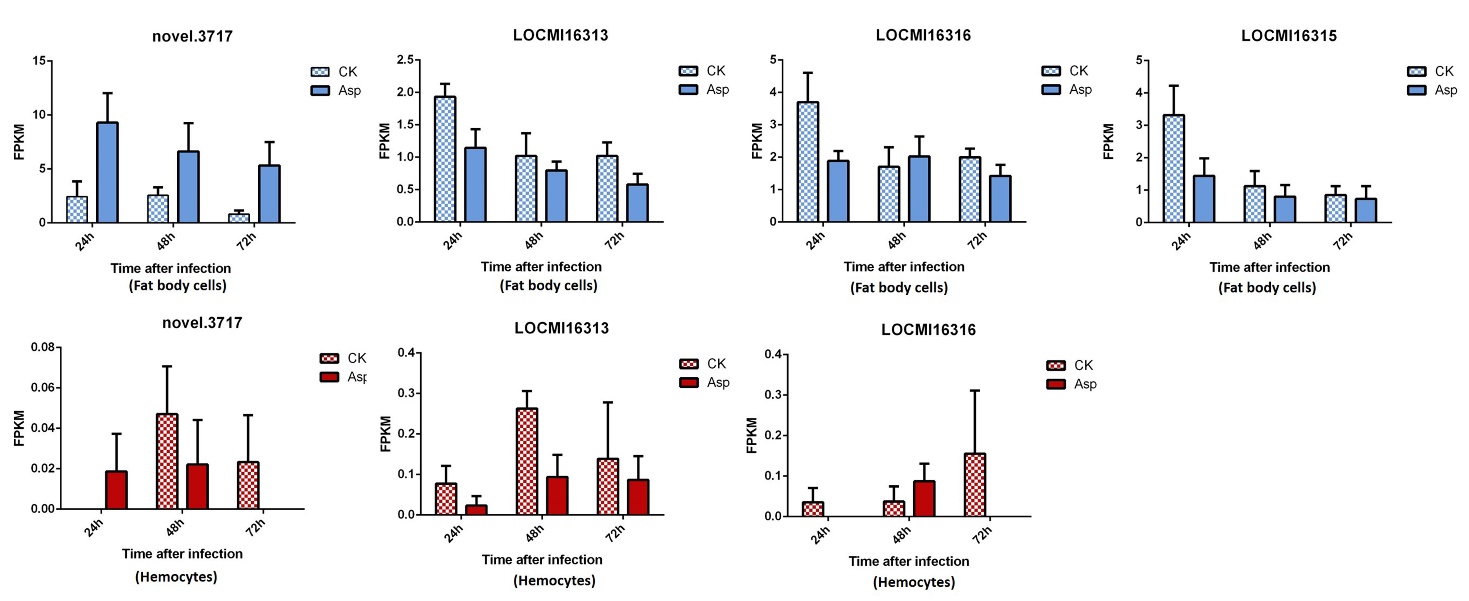
Supplemental Figure 1. Differential statistical analysis of expression levels of the Spätzle genes in fat body and hemocytes between control and infected locusts.** *t*-test. n=3-4. Bar, S.E.M. FFM, female fat body; HFM, Female haemolymph. CK, control. Asp, *A. oryzae*.
